# Monophosphoryl Lipid A–based Adjuvant to Promote the Immunogenicity of Multivalent Meningococcal Polysaccharide Conjugate Vaccines

**DOI:** 10.4049/immunohorizons.2400013

**Published:** 2024-04-16

**Authors:** Kishore R. Alugupalli

**Affiliations:** Department of Microbiology and Immunology, Sidney Kimmel Medical College, Thomas Jefferson University, Philadelphia, PA; and TurboVax Inc., Philadelphia, PA

## Abstract

Activation of the adaptive immune system requires the engagement of costimulatory pathways in addition to B and T cell Ag receptor signaling, and adjuvants play a central role in this process. Many Gram-negative bacterial polysaccharide vaccines, including the tetravalent meningococcal conjugate vaccines (MCV4) and typhoid Vi polysaccharide vaccines, do not incorporate adjuvants. The immunogenicity of typhoid vaccines is due to the presence of associated TLR4 ligands in these vaccines. Because the immunogenicity of MCV4 is poor and requires boosters, I hypothesized that TLR4 ligands are absent in MCV4 and that incorporation of a TLR4 ligand–based adjuvant would improve their immunogenicity. Consistent with this hypothesis, two Food and Drug Administration–approved MCV4 vaccines, MENVEO and MenQuadfi, lack TLR4 ligands. Admixing monophosphoryl lipid A, a TLR4 ligand–based adjuvant formulation named “Turbo” with MCV4 induced significantly improved IgM and IgG responses to all four meningococcal serogroup polysaccharides in adult and aged mice after a single immunization. Furthermore, in infant mice, a single booster was sufficient to promote a robust IgG response and 100% seroconversion when MCV4 was adjuvanted with Turbo. Turbo upregulated the expression of the costimulatory molecules CD40 and CD86 on B cells, and Turbo-driven adjuvanticity is lost in mice deficient in CD40 and CD86. These data suggest that Turbo induces the required costimulatory molecules for its adjuvant activity and that incorporation of Turbo could make bacterial polysaccharide vaccines more immunogenic, minimize booster requirements, and be cost-effective, particularly for those individuals in low- and middle-income and disease-endemic countries.

## Introduction

*Neisseria meningitidis* (meningococcus) is a Gram-negative bacterium that can cause septicemia and meningitis in infants under 1 y of age, children, and young adults and is associated with high morbidity and mortality (1–4). The onset of this disease is rapid, and the case fatality rates range between 10% and 20%, despite available antibiotic treatments. Among survivors, 10–20% develop long-term sequelae, including hearing loss, loss of limbs, skin scarring, and neurodevelopmental deficits, and up to 36% develop deficits in physical, cognitive, or psychological functioning (5, 6). There are 13 meningococcal serogroups based on the structurally distinct capsular polysaccharide expressed by meningococcus. Serogroups A, B, C, W, X, and Y are the most common disease-causing variants, and incidence of the disease and the distribution of serogroups vary by region and the age of the individual (2, 3).

Meningococcal infections are vaccine preventable (2). Currently, two quadrivalent meningococcal polysaccharide conjugate vaccines (MCV4), namely MENVEO and MenQuadfi, covering A, C, W, and Y serogroups (7–9), and two protein-based vaccines covering serogroup B (MenB) (10, 11) are available in the United States. Although MCV4 is effective, a single dose does not provide sufficient protection against meningococcal disease 3–8 y after vaccination (12, 13). Consistent with this, a decrease in serum bactericidal Ab levels occurs by 3–5 y postvaccination (14, 15). The estimates of vaccine efficacy from a single immunization prompted the Advisory Committee on Immunization Practices to add a booster dose of MCV4 for young adults (13, 16). In fact, in infants, the induction of an optimal Ab response by MCV4 requires three boosters at ages 4, 6, and 12 mo, and this is currently a common practice in the United States and several other countries (17). MCV4 is a very costly vaccine even for high-income countries (18–20) and is not an affordable vaccine for the national immunization programs in low- and middle-income countries, particularly in the African meningitis belt region of sub-Saharan countries (21). Following the success of a monovalent serogroup A vaccine, MenAfriVac, in sub-Saharan countries (22), a pentavalent MCV covering serogroups A, C, W, X, and Y is currently advancing through early stages of clinical trials (23). The future success of these vaccines will depend not only on their immunogenicity and efficacy but also on cost and the ever-changing epidemiology of invasive meningococcal diseases in other African countries.

Several factors contributing to the immunogenicity of MCV have been analyzed, but very little has been explored on the role of adjuvants to enhance the immune response to these vaccines (24). In fact, none of the meningococcal subunit vaccines incorporate adjuvants. It is known that Ags alone cannot induce an efficient Ab response (1), and therefore adjuvants are required to improve the immunogenicity of vaccines and facilitate the development of new vaccine formulations. Activation of the adaptive immune system requires the engagement of costimulatory signaling pathways in addition to BCR and TCR signaling, and adjuvants play a central role in this process (25). Stimulation of TLRs with appropriate ligands triggers the induction of costimulatory molecules, amplifies B cell activation, promotes dendritic cell maturation, and increases Ag presentation to T cells (26–28). TLR ligands help direct adaptive immune responses to Ags, and several vaccines incorporate TLR ligands as adjuvants to augment Ag-specific responses (29). The adjuvant effect of a TLR7 agonist adsorbed on aluminum hydroxide (AS37)-adjuvanted meningococcal C conjugated vaccine has been evaluated in a clinical trial (30). The adjuvant effect of AS37 on this monovalent MCV serogroup C vaccines was not significantly different from the unadjuvanted groups (30). Analysis of the immune responses induced by the AS37-adjuvanted vaccine revealed a specific activation of the IFN-mediated antiviral response (31), suggesting that this adjuvant is more suitable for viral vaccines than bacterial polysaccharide vaccines. Indeed, it was shown recently that a TLR7-nanoparticle adjuvant promotes a broad immune response against heterologous strains of influenza and SARS-CoV-2 (32). TLR4 ligands such as lipid A are a signature component of Gram-negative bacterial LPS and were shown to contribute to the immunogenicity of typhoid Vi polysaccharide vaccines (33). As such, in the present study, I tested whether incorporating a nontoxic TLR4 ligand monophosphoryl lipid A (MPLA)-based adjuvant named “Turbo” in MCV4 vaccines would make those vaccines more immunogenic across all ages, regardless of the intrinsic immunogenicity of serogroup polysaccharides or the carrier protein used to make these conjugate vaccines.

## Materials and Methods

### Mice

The Thomas Jefferson University Institutional Animal Care and Use Committee has approved these studies. Mice were housed in microisolator cages with free access to food and water and were maintained in a specific pathogen-free facility. Wild-type C57BL/6J (stock no. 000664), TLR4^−/−^ (stock no. 029015), MyD88^−/−^ (stock no. 009088), CD40^−/−^ (stock no. 002928), and CD86^−/−^ (stock no. 036705) mice on a C57BL/6 background were purchased from The Jackson Laboratory (Bar Harbor, ME). Age-matched mice of both sexes were used for all experiments.

### Adjuvant, Ags, and immunization

The adjuvant named “Turbo” was prepared by mixing 1 mg phosphorylated hexaacyldisaccharide (PHAD), a synthetic MPLA, and 2 mg 1,2-dipalmitoyl-sn-glycero-3-phosphocholine (purchased from Avanti Polar Lipids, Alabaster, AL) in chloroform. Following chloroform evaporation, the contents were suspended in 1% polyethylene glycol sorbitan monooleate (Tween 80; purchased from Sigma-Aldrich) to a concentration of 500 µg/ml MPLA and homogenized by sonication. The homogenate was mixed 50 times using two syringes connected via a 25-gauge needle and passed five times through a polyether sulfone membrane filter with pore size 0.22 µm (Millipore) and stored at 4°C. Nanoparticle tracking analysis using NanoSight NS300 instrumentation (Malvern Instruments, Malvern, UK) revealed that the size distribution and concentration of liposomes in the adjuvant formulation were 130 ± 40 nm and 4 × 10^10^/ml, respectively. The physical characteristics and adjuvant activity was stable for at least 1 y at 4°C.

Quadrivalent meningococcal ACWY polysaccharide conjugate vaccines (MCV4), MENVEO (lot AMVA977A; GlaxoSmithKline) and MenQuadfi (lot U7596AC; Sanofi Pasteur), were purchased. The serogroup A, C, Y, and W-135 polysaccharides of MENVEO and MenQuadfi are conjugated to carrier protein CRM197, a nontoxic mutant protein of diphtheria toxin and tetanus toxoid, respectively.

Adult mice 10–12 wk of age or aged mice 102 wk of age were immunized i.m. with 2.5 µg MENVEO (containing 1 µg A, 0.5 µg C, 0.5 µg W135, and 0.5 µg Y serogroup polysaccharides) or 4 µg MenQuadfi (containing 1 µg A, C, and, W135Y serogroup polysaccharides) admixed with or without Turbo adjuvant (containing 5 µg MPLA) in 50-µl volume in the thigh region of the hind limb. Infant mice (9 d old) were immunized s.c. with 1.25 µg MENVEO or 2 µg MenQuadfi admixed with or without Turbo adjuvant (containing 2.5 µg MPLA) in 25-µl volume in the scruff area between neck and shoulder. The infant mouse group was reimmunized 15 d later, and the adult mouse group was reimmunized 21 d later. Blood samples were obtained as indicated following immunization and stored at −20°C.

### ELISA

Unconjugated serotype polysaccharides MenA (13/262), MenC (08/214), MenW (16/152), or MenY (16/206) were purchased from the National Institute for Biological Standards and Control (Hertfordshire, UK). Serotype polysaccharide-specific IgM and IgG were measured by coating 96-well microtiter plates (Nunc MaxiSorp; Invitrogen, Carlsbad, CA) with 1 µg/ml MenA, MenC, MenW, or MenY in PBS, pH 7.2, overnight at room temperature. All plates were washed and blocked with 1% BSA in PBS, pH 7.2 (blocking buffer), for 2 h at room temperature. Blood from immunized infant and adult mice was diluted to 1:25 and 1:50, respectively, and serogroup-specific IgM and IgG levels were interpreted as ng/µl “equivalents” using normal mouse serum standards (Bethyl Laboratories, Montgomery, TX), mouse isotype–specific capture Abs, and HRP-conjugated anti-mouse IgM, IgG, IgG1, IgG2b, IgG2c, and IgG3 as described previously (33, 34).

### TLR, NOD1, and NOD2 ligand screening

TLR/NOD-like receptor (NLR) ligand screening was performed using a customized PRR ligand screening service offered by InvivoGen (San Diego, CA) (www.invivogen.com/custom-tlr-screening). This screen employs HEK293 cells stably expressing a single human or mouse TLR2, 3, 4, 5, 7, 8, 9, NOD1, or NOD2. Stimulation of these TLRs/NLRs was quantified by NF-κB activation, which induces a secreted embryonic alkaline phosphatase (SEAP) reporter. Reagents were tested in triplicate compared with control ligands in a 96-well plate (200 µl total volume) containing the appropriate cells (50,000–75,000 cells/well). A quantity of 20 µl MENVEO or MenQuadfi vaccines containing 5 µg/ml ACWY serogroup polysaccharides or negative and positive control ligand was added. The media containing Quanti-Blue reagent detects NF-κB induced SEAP expression. OD was read at 650 nm after 16–24 h on a Molecular Devices SpectraMax 340PC absorbance detector.

### Flow cytometry

A total of 3 × 10^6^ wild-type mouse spleen cells/well were plated in a tissue culture–treated, nonpyrogenic, polystyrene 12-well plate in 3 ml culture medium (RPMI 1640 with 25 mM HEPES and l-glutamine + 10% heat-inactivated FBS and penicillin/streptomycin) containing various concentrations of Turbo (containing 2, 0.4, or 0.08 µg MPLA/ml). At 24 h after stimulation, the cells were collected and washed in staining medium (PBS with 3% new calf serum, 1 mM EDTA). Cell concentration was adjusted to 2.0 × 10^7^/ml in staining medium (PBS with 3% new calf serum, 1 mM EDTA). After blocking Fc receptors with 2.4G2 Ab (1 µg per 10^6^ cells), an aliquot of 50 µl cultured cells was incubated with appropriately diluted Abs. The Abs anti-B220-FITC (clone RA3-6B2), anti-B220-BV605 (clone RA3-6B2), anti-CD40-PE (clone 1C10), anti-CD86-PE-Cy7 (clone GL1), anti-CD80-Percp-Cy5.5 (clone 16-10A1), and anti-I-A^b^(MHC-II)-BV421 (clone AF6-120.1) were purchased from BioLegend (San Diego, CA). After staining, cells were washed twice with staining medium, and the preparations were analyzed on a BD FACSymphony (Becton Dickinson, Mountain View, CA) using FACS Diva software (Becton Dickinson). Data were analyzed using the FlowJo software program (FlowJo LLC, Ashland, OR).

### Statistical analysis

Data presented throughout depict pooled data from at least two independent experiments. Statistical analyses were performed using the Prism 10 software program (GraphPad Software, La Jolla, CA), and the statistical tests are indicated in the figure legends.

## Results

### MCV4 vaccines MENVEO and MenQuadfi do not stimulate mouse or human TLR4

**FIGURE 1.**
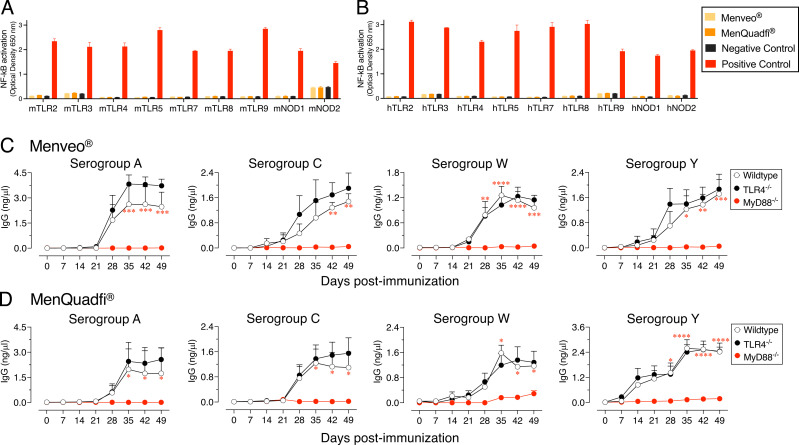
Meningococcal vaccines MENVEO and MenQuadfi do not stimulate TLR4, and their immunogenicity is not dependent on TLR4 signaling. (**A** and **B**) HEK293 cells expressing a given TLR or NLR with an NF-κB–inducible SEAP reporter gene were incubated with MENVEO (5 µg lot AMVA977A/AMXA977A from GlaxoSmithKline) or MenQuadfi (5 µg lot 47643AA from Sanofi Pasteur). Positive controls were 10^8^ heat-killed *Listeria monocytogenes* cells/ml for m/hTLR2, high molecular weight polyinosinic:polycytidylic acid at 1 μg/ml for m/hTLR3, *E. coli* K12 LPS at 100 ng/ml for m/hTLR4, *S. typhimurium* flagellin at 100 ng/ml m/hTLR5, CL307 at 1 μg/ml m/hTLR7, TL8-506 at 1 μg/ml for hTLR8, TL8-506 at 10 μg/ml for mTLR8, CpG oligodeoxynucleotide 2006 at 10 μg/ml for hTLR9, CpG oligodeoxynucleotide 1826 at 1 μg/ml for mTLR9, C12-iE-DAP at 10 μg/ml for m/hNOD1, and L18-MDP at 1 μg/ml m/hNOD1. HEK-Blue null cell lines incubated with the above agonists served as negative controls. The medium containing HEK-Blue Detection is designed for the detection of NF-κB–induced SEAP expression. After a 16–24-h incubation, the OD was read at 650 nm. (**C** and **D**) Wild-type (C57BL/6J), TLR4^−/−^, or MyD88^−/−^ (*n* = 6) male and female mice 8–10 wk of age were immunized i.m. with 50 µl of (C) MENVEO or (D) MenQuadfi vaccine containing 2.5 µg or 4 µg, respectively, of serogroup polysaccharides A, C, W, and Y. On day 21, mice were reimmunized in the same manner. Serogroup-specific IgG levels were measured by ELISA. Statistical analyses were done using two-way ANOVA with Sidak’s multiple comparisons test, and statistically significant differences are indicated as ****p < 0.0001; ***p < 0.001; **p < 0.001; *p < 0.05.

The immunogenicity of typhoid Vi polysaccharide subunit vaccines (e.g., Typhim Vi and Typbar TCV) is dependent on the “adjuvant-like” activity of TLR4 ligands present in them (33). Unlike the typhoid vaccines, MENVEO and MenQuadfi are not very immunogenic, suggesting that these vaccines do not contain associated TLR4 or other ligands, such as bacterial peptidoglycan fragments that are recognized by NOD1 and NOD2. To test the presence of these ligands in MCV4 vaccines, a panel of mouse or human TLR2-, TLR3-, TLR4-, TLR5-, TLR7-, TLR8-, TLR9-, NOD1-, and NOD2-expressing cells with an NF-κB reporter system were screened. In this screen, neither MENVEO nor MenQuadfi activated any of the mouse or human TLRs, NOD1, or NOD2 (Fig. 1A, 1B).

### The immunogenicity of MENVEO and MenQuadfi is dependent on MyD88 but not on TLR4

To test a potential role for TLR4 or other TLRs in MCV4 vaccine immunogenicity in vivo, wild-type, TLR4^−/−^, or mice deficient in MyD88, an adaptor required for signaling of all TLRs (except TLR3) and IL-1/18R, were immunized with MENVEO and MenQuadfi. Consistent with the lack of TLR4 stimulatory activity in these vaccines (Fig. 1A, 1B), the IgG responses in wild-type and TLR4^−/−^ mice were comparable (Fig. 1C, 1D). On the other hand, a severe impairment of IgG responses to all four serogroup polysaccharides was observed in MyD88^−/−^ mice (Fig. 1C, 1D), indicating that the immunogenicity of MCV4 vaccines is dependent on the MyD88 signaling axis and suggesting that trace amounts of other TLR ligands (below the detection limits of the above in vitro assay) in MCV4 may be playing a role in MCV4 immunogenicity in vivo.

### The immunogenicity of MENVEO or MenQuadfi is promoted by Turbo as an adjuvant in adult and aged mice

**FIGURE 2.**
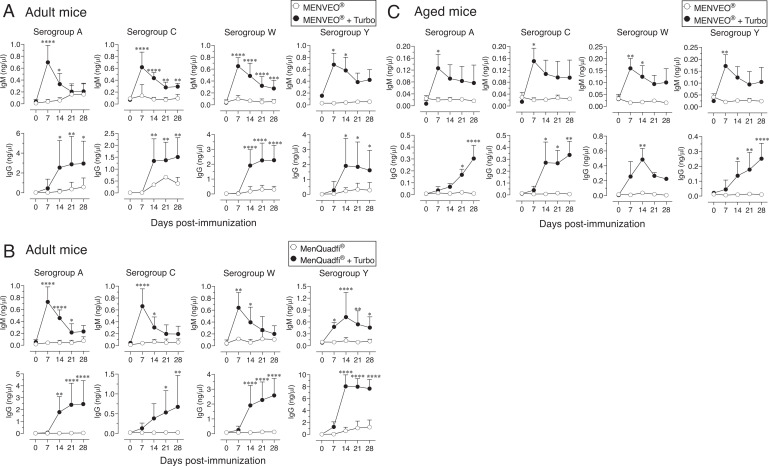
The immunogenicity of meningococcal vaccine is enhanced by Turbo in adult and aged mice. C57BL/6J mice 12 wk (*n* = 8) or 102 wk (*n* = 4) of age were immunized i.m. with 50 µl (**A**and **C**) MENVEO or (**B**) MenQuadfi vaccine containing 2.5 µg or 4 µg, respectively, of serogroup polysaccharides A, C, W, and Y admixed with or without Turbo (5 µg MPLA), and serogroup-specific IgM and IgG levels were measured by ELISA. Statistical analyses were done using two-way ANOVA and Sidak’s multiple comparisons test, and statistically significant differences are indicated as ****p < 0.0001; ***p < 0.001; **p < 0.001; *p < 0.05.

In young adult mice (e.g., 8–12 wk of age), at least one booster dose is required to detect an appreciable IgG response to MCV4, regardless of the mouse strain used or whether an s.c. or i.p. route of immunization is used (35). We confirmed this observation with i.m. immunization as well (Fig. 1C, 1D). To test whether incorporation of an adjuvant would eliminate the need for a booster immunization, MENVEO and MenQuadfi were admixed with Turbo. I found that robust IgM and IgG responses in adult mice were observed for both MCV4 vaccines when adjuvanted with Turbo with a single immunization, unlike that with unadjuvanted MCV4 vaccines (Fig. 2A, 2B).

Aged individuals do not respond efficiently to vaccines due to immune senescence. Studies in mice have shown that the impairment in immune responses in aged mice is due to several parameters, including a decreased number of APCs and their interaction with T cells (36, 37) and an increased threshold required for TLR signaling (38). However, when MENVEO was adjuvanted with Turbo, aged mice showed a significant improvement in IgM and IgG responses to all four serogroup polysaccharides of MCV4 with a single immunization (Fig. 2C).

### Admixing Turbo enhances the immunogenicity of MENVEO and MenQuadfi in infant mice

**FIGURE 3.**
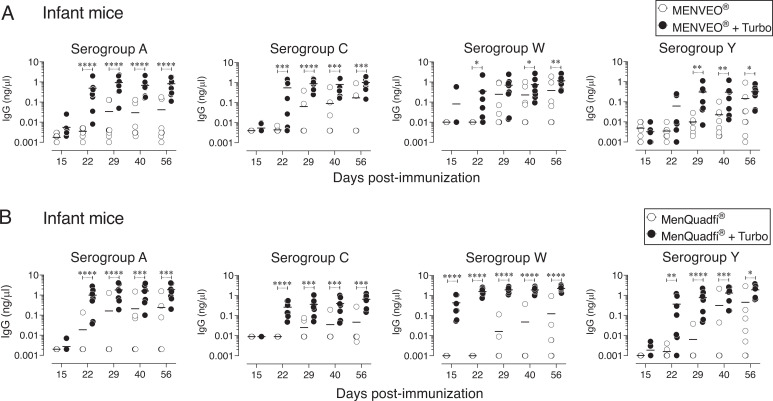
Turbo enhances the immunogenicity of MENVEO and MenQuadfi in infant mice. Groups of —seven or eight infant mice of both sexes (9 d old) were immunized s.c. with (**A**) 1.25 µg MENVEO or (**B**) 2 µg MenQuadfi admixed (black circles) or without (white circles) Turbo adjuvant (2.5 µg MPLA) in 25-µl volume in the scruff area between the neck and shoulder. On day 15, mice were reimmunized in the same manner. Serogroup-specific IgG levels were measured by ELISA. Each dot represents an individual mouse, and the black bar represents the mean. Statistics were determined by Mann–Whitney *U* test, and statistically significant differences are indicated as ****p < 0.0001; ***p < 0.001; **p < 0.001; *p < 0.05.

The lack of an efficient Ab response to polysaccharide vaccines in human infants and infant mice in part is due to incomplete B cell development and a restricted B cell Ag receptor repertoire (39–42). Therefore, to generate optimal Ab responses in infants, multiple immunizations at 2, 4, 6, and 12 mo of age are required. Because neither of the MCV4 vaccines has endogenous TLR4 ligand activity (Fig. 1), MENVEO and MenQuadfi were admixed with Turbo. I found that a single prime-boost strategy dramatically increased the immunogenicity of these vaccines when adjuvanted with Turbo (Fig. 3). Compared with unadjuvanted MCV4 vaccines, seroconversion of 100% was achieved against all four serogroup-specific polysaccharides when adjuvanted with Turbo (Fig. 3). These data (Figs. 2 and 3) indicate that Turbo as an adjuvant improves the MCV4 responses across all ages.

### Turbo upregulates the expression of CD86 and MHC-II on B cells, and Turbo adjuvanticity is dependent on CD86 and CD40

**FIGURE 4.**
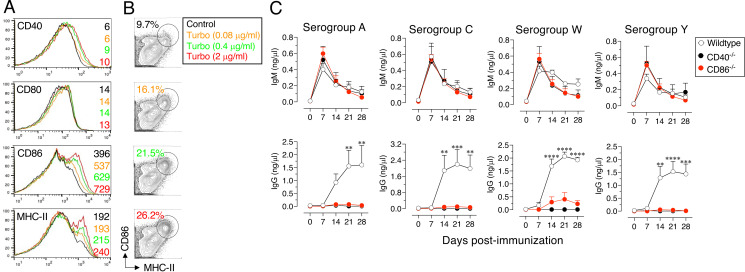
Turbo upregulates the expression of CD40, CD86, and MHC-II on B cells, and Turbo adjuvanticity is dependent on CD40 and CD86 in vivo. Spleen cells of naive C57BL/6 mice were incubated without or with various concentrations of Turbo for 18–24 h. All cells were stained with specific Abs and subjected to flow cytometric analysis. (**A**) The surface expression of CD40, CD80, CD86, and MHC-II on B cells (gated as CD19- or B220-positive and B220 double-positive) was measured. Geometric mean fluorescence intensity values matching Turbo concentration (color coded) are indicated within each plot. (**B**) The frequency of B cells that express high levels of both CD86 and MHC-II among all B cells from control and Turbo treatment groups is shown. The data were generated by analyzing a minimum of 200,000 cells and 5% contour plots are shown. (**C**) Wild-type (C57BL/6J), CD40^−/−^, or CD86^−/−^ (*n* = 4) male and female mice 8–10 wk of age were immunized i.m. with 50 µl of MENVEO (1.25 µg) admixed with Turbo adjuvant (2.5 µg MPLA). Serogroup-specific IgM and IgG levels were measured by ELISA. Statistical analyses were done using two-way ANOVA with Sidak’s multiple comparisons test, and statistically significant differences are indicated as ****p < 0.0001; ***p < 0.001; **p < 0.001.

Initiation of adaptive immune responses involves the engagement of the costimulatory molecules CD80 and CD86 in addition to B and T cell Ag receptor signaling (25). The expression of CD40 and MHC-II on B cells is required for sustaining the germinal center reaction, which is central to T cell–dependent B cell responses (43). Stimulation of B cells with LPS upregulates the surface expression of CD40, CD80, and CD86 as well as MHC-II to various levels (27, 28, 43). Because MCV4 induces a T cell–dependent B cell response (44) and Turbo enhances the Ab response to MCV4 across all ages (Figs. 2 and 3), using a cell culture assay, I tested whether Turbo induces the upregulation of CD40, CD80, CD86, and MHC-II on B cells. I found that treatment of spleen cells with Turbo increases expression of CD86 and MHC-II on B cells (Fig. 4A). The frequency of CD86^hi^/MHC-II^hi^ B cells was also increased in a dose-dependent manner (Fig. 4B). Although the expression of CD80 was not altered, there was a noticeable increase in CD40 expression on B cells treated with Turbo in this 24-h in vitro culture assay (Fig. 4A). To test the role of CD40 and CD86 in Turbo adjuvanticity in vivo, wild-type mice and mice deficient in CD40 or CD86 were immunized with MENVEO adjuvanted with Turbo, and Ab responses to A, C, W, and Y serogroup polysaccharides were analyzed. Although IgM responses were similar among all mouse groups, unlike wild-type mice, CD86^−/−^ and CD40^−/−^ mice exhibited a severe impairment in generating IgG responses to all four serogroup polysaccharides (Fig. 4C). These data suggest that the adjuvanticity of Turbo appears in part to be due to upregulation of costimulatory receptors.

## Discussion

The central idea of coupling a bacterial polysaccharide Ag to a protein carrier such as tetanus or diphtheria toxoids (i.e., conjugate vaccines) is to make polysaccharide Ags immunogenic for infants. This modification converts T cell–independent polysaccharide Ags into bona fide T cell–dependent B cell Ags (44). In this process, polysaccharide-recognizing B cells also act as APCs because they can process the internalized carrier protein and present the resultant peptides on MHC-II to T cells to receive their help (44). However, when the TCR engages peptide-MHC-II in the absence of costimulatory molecules, T cells become anergic and remain unresponsive even when such T cells are restimulated with peptide-MHC-II and a costimulatory signal (45). Although B cells can respond to unconjugated polysaccharides in adults primarily by their BCR crosslinking (46, 47), if those B cells do not receive a secondary signal, they die by activation-induced mitochondrial dysfunction (48–50). Therefore, a second signal is a requirement for initiation of both B and T cell responses, and thus vaccines containing only Ags without adjuvants that provide the second signal are not expected to initiate an efficient response. Surprisingly, most Gram-negative bacterial polysaccharide vaccines, including MCV4 and those against *Haemophilus influenzae* type B e.g., ActHIB and HIBERIX or *Salmonella* Typhi e.g., Typhim Vi and Typbar TCV, do not incorporate adjuvants, and their immunogenicity varies dramatically from vaccine to vaccine. For example, unlike MCV4, typhoid vaccines induce an efficient response with a single immunization even in infants (51–53). Because the polysaccharide Ags used to make these bacterial vaccines are directly isolated from their respective bacterial species, I previously hypothesized that these vaccines may contain other bacterial components, such as TLR ligands, that could act as adjuvants. Indeed, analysis of immunogenic Typhim Vi and Typbar TCV revealed that these vaccines contain endotoxin/LPS TLR4 stimulators, and their immunogenicity is largely due to the presence of these TLR ligands (33). Although the serogroup A, C, W, and Y polysaccharides are also isolated directly from meningococcus, a Gram-negative bacterium, to make MCV4, they do not appear to contain detectable levels of TLR4 ligands (Fig. 1A, 1B), unlike the high levels seen in typhoid Vi polysaccharide vaccines (33). In fact, unlike the typhoid vaccine response after a single immunization in infants (51–53), the induction of an optimal Ab response by many other polysaccharide vaccines, including MCV4 and *Haemophilus influenzae* type B, require first immunization at 2 mo of age and three boosters at ages 4, 6, and 12–15 mo. This difference in the vaccine-associated TLR ligand activity could account for the poor immunogenicity of MCV4 compared with typhoid vaccines. However, the complete lack of an Ab response in vivo to MCV4 in mice deficient in MyD88, an adaptor required for signaling through several members of the TLR family, suggests that trace amounts of other TLR ligands (which are below the detection limit of the in vitro assay in Fig. 1A, 1B) in MENVEO and MenQuadfi contribute to the immunogenicity of MCV4 (Fig. 1C, 1D). Despite their safety across all ages, the major disadvantage of subunit vaccines that do not include adjuvants is their poor immunogenicity. The present study shows that incorporation of a TLR4 ligand-based adjuvant such as Turbo helps overcome this poor immunogenicity, as shown by the significantly improved Ab response and seroconversion with a single booster in infant mice and elimination of the need for a booster in adult mice.

Many TLR ligands, including LPS/lipid A, can upregulate costimulatory receptors such as CD86 as well as MHC-II on murine B cells (27, 28). Consistent with this, I show that Turbo containing MPLA, a nontoxic derivative of lipid A and a 1000-fold less potent TLR4 ligand than LPS/lipid A (54), increases the levels of both MHC-II and CD86 on B cells (Fig. 4A, 4B). Mice deficient in CD86 despite having normal CD80 (43) did not generate an Ab response to MCV4 (Fig. 4C), indicating that CD86 is part of the mechanism by which Turbo acts as an adjuvant. CD40 is known to upregulate the expression of activation-induced cytidine deaminase (AID), which is required for Ig class switching and affinity maturation. The expression of AID is induced by BCR and TLR stimulation even in the absence of T cell help (55–57). Unlike CD86 expression, CD40 expression induced by LPS stimulation is low on B cells (28). Consistent with this, the upregulation of CD40 by Turbo is marginal (Fig. 4A). Nevertheless, CD40^−/−^ mice, when immunized with Turbo-admixed MENVEO, failed to generate an IgG response (Fig. 4B), suggesting that in addition to CD86, CD40 is also required for Turbo adjuvanticity in vivo. These two costimulatory molecules act at distinct phases of Ab responses. The CD86-mediated costimulation is required for the initial B and T cell activation, and CD40 signaling plays an important role in the downstream phase of activation, such as the germinal center reaction. It is known that germinal center B and T follicular helper cell interactions result in CD40-CD40L–driven AID expression and IgM class switching to IgG isotypes. Consistent with this, the short-lived IgM response, which is induced primarily by BCR crosslinking by Ags containing repetitive epitopes such as polysaccharides, is comparable in wild-type and CD40^−/−^ or CD86^−/−^ mice. Therefore, adjuvants containing TLR4 ligands such as MPLA are expected to promote long-lived IgG Ab responses to both unconjugated and conjugated polysaccharide vaccines, as shown with typhoid vaccines (K. R. Alugupalli, manuscript posted on bioRxiv, DOI: 10.1101/2024.03.22.581918).

The inclusion of MPLA as an adjuvant component has already been approved by the Food and Drug Administration (FDA) for a few viral vaccines (e.g., zoster vaccine [Shingrix], human papillomavirus vaccine [Cervarix], and hepatitis B vaccine [Fendrix]). In addition to MPLA, these vaccines also contain other adjuvant components, such as QS21, a fraction extracted from *Quillaja saponaria* plant, and Al(OH)_3_ or AlPO_4_, commonly referred to as alum. Therefore, the impact of MPLA alone in these vaccines remains to be determined. In contrast to the above examples, the present study demonstrates that incorporating an MPLA alone–based adjuvant formulation named “Turbo” into two FDA-approved multivalent MCV4 vaccines generated significantly improved IgM and IgG responses to all four meningococcal serogroup polysaccharides across all ages. Therefore, admixing Turbo containing MPLA at the same doses used in the above-mentioned FDA-approved vaccines into MCV4 or other multivalent vaccines against *Escherichia coli* (58), *Shigella* (59), and *Salmonella* (60) that are undergoing a development phase could make bacterial polysaccharide vaccines more immunogenic and efficacious and could increase the compliance associated with minimizing boosters. This would therefore make such vaccines cost-effective, particularly for individuals in low- and middle-income countries.

## References

[1] Wang, B., R. Santoreneos, L. Giles, H. Haji Ali Afzali, H. Marshall. 2019. Case fatality rates of invasive meningococcal disease by serogroup and age: a systematic review and meta-analysis. Vaccine 37: 2768–2782. 30987851 10.1016/j.vaccine.2019.04.020

[2] Pelton, S. I. 2016. The global evolution of meningococcal epidemiology following the introduction of meningococcal vaccines. J. Adolesc. Health 59(2 Suppl): S3–S11. 27449148 10.1016/j.jadohealth.2016.04.012

[3] Caugant, D. A., O. B. Brynildsrud. 2020. *Neisseria meningitidis*: using genomics to understand diversity, evolution and pathogenesis. Nat. Rev. Microbiol. 18: 84–96. 31705134 10.1038/s41579-019-0282-6

[4] Rosenstein, N. E., B. A. Perkins, D. S. Stephens, T. Popovic, J. M. Hughes. 2001. Meningococcal disease. N. Engl. J. Med. 344: 1378–1388. 11333996 10.1056/NEJM200105033441807

[5] Viner, R. M., R. Booy, H. Johnson, W. J. Edmunds, L. Hudson, H. Bedford, E. Kaczmarski, K. Rajput, M. Ramsay, D. Christie. 2012. Outcomes of invasive meningococcal serogroup B disease in children and adolescents (MOSAIC): a case-control study. Lancet Neurol. 11: 774–783. 22863608 10.1016/S1474-4422(12)70180-1

[6] Vyse, A., A. Anonychuk, A. Jakel, H. Wieffer, S. Nadel. 2013. The burden and impact of severe and long-term sequelae of meningococcal disease. Expert Rev. Anti Infect. Ther. 11: 597–604. 23750731 10.1586/eri.13.42

[7] Jackson, L. A., R. Baxter, K. Reisinger, A. Karsten, J. Shah, L. Bedell, P. M. Dull; V59P13 Study Group. 2009. Phase III comparison of an investigational quadrivalent meningococcal conjugate vaccine with the licensed meningococcal ACWY conjugate vaccine in adolescents. Clin. Infect. Dis. 49: e1–e10. 19476428 10.1086/599117

[8] Cooper, B., L. DeTora, J. Stoddard. 2011. Menveo: a novel quadrivalent meningococcal CRM197 conjugate vaccine against serogroups A, C, W-135 and Y. Expert Rev. Vaccines 10: 21–33. 21162617 10.1586/erv.10.147

[9] Huston, J., K. Galicia, E. F. Egelund. 2022. MenQuadfi (MenACWY-TT): a new vaccine for meningococcal serogroups ACWY. Ann. Pharmacother. 56: 727–735. 34459258 10.1177/10600280211039873

[10] Santolaya, M. E., M. L. O’Ryan, M. T. Valenzuela, V. Prado, R. Vergara, A. Munoz, D. Toneatto, G. Grana, H. Wang, R. Clemens, V72P10 Meningococcal B Adolescent Vaccine Study Group. 2012. Immunogenicity and tolerability of a multicomponent meningococcal serogroup B (4CMenB) vaccine in healthy adolescents in Chile: a phase 2b/3 randomised, observer-blind, placebo-controlled study. Lancet 379: 617–624. 22260988 10.1016/S0140-6736(11)61713-3

[11] Beeslaar, J., J. Absalon, P. Balmer, A. Srivastava, R. Maansson, L. J. York, J. L. Perez. 2018. Clinical data supporting a 2-dose schedule of MenB-FHbp, a bivalent meningococcal serogroup B vaccine, in adolescents and young adults. Vaccine 36: 4004–4013. 29861182 10.1016/j.vaccine.2018.05.060

[12] Gill, C. J., R. Baxter, A. Anemona, G. Ciavarro, P. Dull. 2010. Persistence of immune responses after a single dose of Novartis meningococcal serogroup A, C, W-135 and Y CRM-197 conjugate vaccine (Menveo) or Menactra among healthy adolescents. Hum. Vaccin. 6: 881–887. 21339701 10.4161/hv.6.11.12849 PMC3060384

[13] Cohn, A. C., J. R. MacNeil, L. H. Harrison, R. Lynfield, A. Reingold, W. Schaffner, E. R. Zell, B. Plikaytis, X. Wang, N. E. Messonnier; Active Bacterial Core Surveillance (ABCs) Team and MeningNet Surveillance Partners. 2017. Effectiveness and duration of protection of one dose of a meningococcal conjugate vaccine. Pediatrics 139: e20162193. 28100689 10.1542/peds.2016-2193 PMC8353579

[14] Baxter, R., K. Reisinger, S. L. Block, A. Izu, T. Odrljin, P. Dull. 2014. Antibody persistence and booster response of a quadrivalent meningococcal conjugate vaccine in adolescents. J. Pediatr. 164: 1409–1415.e4. 24657122 10.1016/j.jpeds.2014.02.025

[15] Jacobson, R. M., L. A. Jackson, K. Reisinger, A. Izu, T. Odrljin, P. M. Dull. 2013. Antibody persistence and response to a booster dose of a quadrivalent conjugate vaccine for meningococcal disease in adolescents. Pediatr. Infect. Dis. J. 32: e170–e177. 23114372 10.1097/INF.0b013e318279ac38

[16] Zambrano, B., J. Peterson, C. Deseda, K. Julien, C. A. Spiegel, C. Seyler, M. Simon, R. Hoki, M. Anderson, B. Brabec, 2023. Quadrivalent meningococcal tetanus toxoid-conjugate booster vaccination in adolescents and adults: phase III randomized study. Pediatr. Res. 94: 1035–1043. 36899125 10.1038/s41390-023-02478-5 PMC10000353

[17] Pinto Cardoso, G., M. Lagree-Chastan, M. Caseris, J. Gaudelus, H. Haas, J. P. Leroy, P. Bakhache, J. F. Pujol, A. Werner, M. A. Dommergues, 2022. Overview of meningococcal epidemiology and national immunization programs in children and adolescents in 8 Western European countries. Front. Pediatr. 10: 1000657. 36507149 10.3389/fped.2022.1000657 PMC9727280

[18] Shepard, C. W., I. R. Ortega-Sanchez, R. D. Scott 2nd, N. E. Rosenstein; ABCs Team. 2005. Cost-effectiveness of conjugate meningococcal vaccination strategies in the United States. Pediatrics 115: 1220–1232. 15867028 10.1542/peds.2004-2514

[19] Kim, J. J. 2011. The role of cost-effectiveness in U.S. vaccination policy. N. Engl. J. Med. 365: 1760–1761. 22010866 10.1056/NEJMp1110539

[20] Watle, S. V., L. M. Næss, G. Tunheim, D. A. Caugant, T. Wisløff. 2021. Cost-effectiveness of meningococcal vaccination of Norwegian teenagers with a quadrivalent ACWY conjugate vaccine. Hum. Vaccin. Immunother. 17: 2777–2787. 33631080 10.1080/21645515.2021.1880209 PMC8475610

[21] Mustapha, M. M., L. H. Harrison. 2018. Vaccine prevention of meningococcal disease in Africa: major advances, remaining challenges. Hum. Vaccin. Immunother. 14: 1107–1115. 29211624 10.1080/21645515.2017.1412020 PMC5989898

[22] Viviani, S. 2022. Efficacy and effectiveness of the meningococcal conjugate group A vaccine MenAfriVac in preventing recurrent meningitis epidemics in sub-Saharan Africa. Vaccines (Basel) 10:10.3390/vaccines10040617 PMC9027557 35455366

[23] Alderson, M. R., F. M. LaForce, A. Sobanjo-Ter Meulen, A. Hwang, M. P. Preziosi, K. P. Klugman. 2019. Eliminating meningococcal epidemics from the African meningitis belt: the case for advanced prevention and control using next-generation meningococcal conjugate vaccines. J. Infect. Dis. 220(Suppl 4): S274–S278. 31671447 10.1093/infdis/jiz297 PMC6822963

[24] Broker, M., F. Berti, P. Costantino. 2016. Factors contributing to the immunogenicity of meningococcal conjugate vaccines. Hum. Vaccin. Immunother. 12: 1808–1824. 26934310 10.1080/21645515.2016.1153206 PMC4964817

[25] Janeway, C. A. Jr. 1989. Approaching the asymptote? Evolution and revolution in immunology. Cold Spring Harb. Symp. Quant. Biol. 54 Pt 1: 1–13. 10.1101/sqb.1989.054.01.003 2700931

[26] Janeway, C. A. Jr., and R. Medzhitov. 2002. Innate immune recognition. Annu. Rev. Immunol. 20: 197–216. 11861602 10.1146/annurev.immunol.20.083001.084359

[27] Yanagibashi, T., Y. Nagai, Y. Watanabe, M. Ikutani, Y. Hirai, K. Takatsu. 2015. Differential requirements of MyD88 and TRIF pathways in TLR4-mediated immune responses in murine B cells. Immunol. Lett. 163: 22–31. 25448706 10.1016/j.imlet.2014.11.012

[28] Xu, H., L. N. Liew, I. C. Kuo, C. H. Huang, D. L. Goh, K. Y. Chua. 2008. The modulatory effects of lipopolysaccharide-stimulated B cells on differential T-cell polarization. Immunology 125: 218–228. 18355243 10.1111/j.1365-2567.2008.02832.x PMC2561127

[29] Steinhagen, F., T. Kinjo, C. Bode, D. M. Klinman. 2011. TLR-based immune adjuvants. Vaccine 29: 3341–3355. 20713100 10.1016/j.vaccine.2010.08.002 PMC3000864

[30] Gonzalez-Lopez, A., J. Oostendorp, T. Koernicke, T. Fadini, U. D’Oro, S. Baker, D. T. O’Hagan, G. Del Giudice, E. Siena, O. Finco, D. Medini. 2019. Adjuvant effect of TLR7 agonist adsorbed on aluminum hydroxide (AS37): a phase I randomized, dose escalation study of an AS37-adjuvanted meningococcal C conjugated vaccine. Clin. Immunol. 209: 108275. 31669193 10.1016/j.clim.2019.108275

[31] Siena, E., F. Schiavetti, E. Borgogni, M. Taccone, E. Faenzi, M. Brazzoli, S. Aprea, M. Bardelli, G. Volpini, F. Buricchi, 2023. Systems analysis of human responses to an aluminium hydroxide-adsorbed TLR7 agonist (AS37) adjuvanted vaccine reveals a dose-dependent and specific activation of the interferon-mediated antiviral response. Vaccine 41: 724–734. 36564274 10.1016/j.vaccine.2022.12.006

[32] Yin, Q., W. Luo, V. Mallajosyula, Y. Bo, J. Guo, J. Xie, M. Sun, R. Verma, C. Li, C. M. Constantz, 2023. A TLR7-nanoparticle adjuvant promotes a broad immune response against heterologous strains of influenza and SARS-CoV-2. Nat. Mater. 22: 380–390. 36717665 10.1038/s41563-022-01464-2 PMC9981462

[33] Alugupalli, K. R. 2024. TLR4 ligands in typhoid Vi polysaccharide subunit vaccines contribute to immunogenicity. Immunohorizons. 8: 29–34. 38180344 10.4049/immunohorizons.2300085 PMC10832388

[34] Pandya, K. D., I. Palomo-Caturla, J. A. Walker, K. S. V, Z. Zhong, K. R. Alugupalli. 2018. An unmutated IgM response to the Vi polysaccharide of *Salmonella* Typhi contributes to protective immunity in a murine model of typhoid. J. Immunol. 200: 4078–4084. 29743315 10.4049/jimmunol.1701348 PMC6033073

[35] Arunachalam, A. B., S. Vile, A. Rosas. 2022. A mouse immunogenicity model for the evaluation of meningococcal conjugate vaccines. Front. Immunol. 13: 814088. 35126397 10.3389/fimmu.2022.814088 PMC8812382

[36] Boraschi, D., M. T. Aguado, C. Dutel, J. Goronzy, J. Louis, B. Grubeck-Loebenstein, R. Rappuoli, G. Del Giudice. 2013. The gracefully aging immune system. Sci. Transl. Med. 5: 185ps8. 10.1126/scitranslmed.3005624 23677590

[37] Tamir, A., M. D. Eisenbraun, G. G. Garcia, R. A. Miller. 2000. Age-dependent alterations in the assembly of signal transduction complexes at the site of T cell/APC interaction. J. Immunol. 165: 1243–1251. 10903722 10.4049/jimmunol.165.3.1243

[38] Panda, A., F. Qian, S. Mohanty, D. van Duin, F. K. Newman, L. Zhang, S. Chen, V. Towle, R. B. Belshe, E. Fikrig, 2010. Age-associated decrease in TLR function in primary human dendritic cells predicts influenza vaccine response. J. Immunol. 184: 2518–2527. 20100933 10.4049/jimmunol.0901022 PMC3867271

[39] Jeong, H. D., J. M. Teale. 1988. Comparison of the fetal and adult functional B cell repertoires by analysis of VH gene family expression. J. Exp. Med. 168: 589–603. 3261774 10.1084/jem.168.2.589 PMC2189009

[40] Malynn, B. A., G. D. Yancopoulos, J. E. Barth, C. A. Bona, F. W. Alt. 1990. Biased expression of JH-proximal VH genes occurs in the newly generated repertoire of neonatal and adult mice. J. Exp. Med. 171: 843–859. 2261012 10.1084/jem.171.3.843 PMC2187788

[41] Schroeder, H. W. Jr., L. Zhang, J. B. Philips 3rd, 2001. Slow, programmed maturation of the immunoglobulin HCDR3 repertoire during the third trimester of fetal life. Blood 98: 2745–2751. 11675347 10.1182/blood.v98.9.2745

[42] Rother, M. B., K. Jensen, M. van der Burg, F. S. van de Bovenkamp, R. Kroek, I. W. F. van, V. H. van der Velden, T. Cupedo, O. K. Olstad, J. J. van Dongen, M. C. van Zelm. 2016. Decreased IL7Ralpha and TdT expression underlie the skewed immunoglobulin repertoire of human B-cell precursors from fetal origin. Sci. Rep. 6: 33924. 27658954 10.1038/srep33924 PMC5034271

[43] Watanabe, M., C. Fujihara, A. J. Radtke, Y. J. Chiang, S. Bhatia, R. N. Germain, R. J. Hodes. 2017. Co-stimulatory function in primary germinal center responses: CD40 and B7 are required on distinct antigen-presenting cells. J. Exp. Med. 214: 2795–2810. 28768709 10.1084/jem.20161955 PMC5584122

[44] Pollard, A. J., K. P. Perrett, P. C. Beverley. 2009. Maintaining protection against invasive bacteria with protein-polysaccharide conjugate vaccines. Nat. Rev. Immunol. 9: 213–220. 19214194 10.1038/nri2494

[45] Parham, P., C. Janeway. 2015. The Immune System. Garland Science, New York.

[46] Vos, Q., A. Lees, Z. Q. Wu, C. M. Snapper, J. J. Mond. 2000. B-cell activation by T-cell-independent type 2 antigens as an integral part of the humoral immune response to pathogenic microorganisms. Immunol. Rev. 176: 154–170. 11043775 10.1034/j.1600-065x.2000.00607.x

[47] Lesinski, G. B., M. A. Westerink. 2001. Novel vaccine strategies to T-independent antigens. J. Microbiol. Methods 47: 135–149. 11576678 10.1016/s0167-7012(01)00290-1

[48] Akkaya, M., S. K. Pierce. 2019. From zero to sixty and back to zero again: the metabolic life of B cells. Curr. Opin. Immunol. 57: 1–7. 30312894 10.1016/j.coi.2018.09.019 PMC6456432

[49] Akkaya, M., J. Traba, A. S. Roesler, P. Miozzo, B. Akkaya, B. P. Theall, H. Sohn, M. Pena, M. Smelkinson, J. Kabat, 2018. Second signals rescue B cells from activation-induced mitochondrial dysfunction and death. Nat. Immunol. 19: 871–884. 29988090 10.1038/s41590-018-0156-5 PMC6202187

[50] Kwak, K., M. Akkaya, S. K. Pierce. 2019. B cell signaling in context. Nat. Immunol. 20: 963–969. 31285625 10.1038/s41590-019-0427-9

[51] Patel, P. D., P. Patel, Y. Liang, J. E. Meiring, T. Misiri, F. Mwakiseghile, J. K. Tracy, C. Masesa, H. Msuku, D. Banda, ; TyVAC Malawi Team. 2021. Safety and efficacy of a typhoid conjugate vaccine in Malawian children. N. Engl. J. Med. 385: 1104–1115. 34525285 10.1056/NEJMoa2035916 PMC8202713

[52] Qadri, F., F. Khanam, X. Liu, K. Theiss-Nyland, P. K. Biswas, A. I. Bhuiyan, F. Ahmmed, R. Colin-Jones, N. Smith, S. Tonks, 2021. Protection by vaccination of children against typhoid fever with a Vi-tetanus toxoid conjugate vaccine in urban Bangladesh: a cluster-randomised trial. Lancet 398: 675–684. 34384540 10.1016/S0140-6736(21)01124-7 PMC8387974

[53] Shakya, M., M. Voysey, K. Theiss-Nyland, R. Colin-Jones, D. Pant, A. Adhikari, S. Tonks, Y. F. Mujadidi, P. O’Reilly, O. Mazur, ; TyVAC Nepal Team. 2021. Efficacy of typhoid conjugate vaccine in Nepal: final results of a phase 3, randomised, controlled trial. Lancet Glob. Health 9: e1561–e1568. 34678198 10.1016/S2214-109X(21)00346-6 PMC8551681

[54] Wang, Y. Q., H. Bazin-Lee, J. T. Evans, C. R. Casella, T. C. Mitchell. 2020. MPL adjuvant contains competitive antagonists of human TLR4. Front. Immunol. 11: 577823. 33178204 10.3389/fimmu.2020.577823 PMC7596181

[55] Pone, E. J., Z. Xu, C. A. White, H. Zan, P. Casali. 2012. B cell TLRs and induction of immunoglobulin class-switch DNA recombination. Front. Biosci. (Landmark Ed.) 17: 2594–2615. 22652800 10.2741/4073 PMC4095906

[56] Pone, E. J., Z. Lou, T. Lam, M. L. Greenberg, R. Wang, Z. Xu, P. Casali. 2015. B cell TLR1/2, TLR4, TLR7 and TLR9 interact in induction of class switch DNA recombination: modulation by BCR and CD40, and relevance to T-independent antibody responses. Autoimmunity 48: 1–12. 25536171 10.3109/08916934.2014.993027 PMC4625915

[57] Rivera, C. E., Y. Zhou, D. P. Chupp, H. Yan, A. D. Fisher, R. Simon, H. Zan, Z. Xu, P. Casali. 2023. Intrinsic B cell TLR-BCR linked coengagement induces class-switched, hypermutated, neutralizing antibody responses in absence of T cells. Sci. Adv. 9: eade8928. 37115935 10.1126/sciadv.ade8928 PMC10146914

[58] Huttner, A., C. Hatz, G. van den Dobbelsteen, D. Abbanat, A. Hornacek, R. Frolich, A. M. Dreyer, P. Martin, T. Davies, K. Fae, 2017. Safety, immunogenicity, and preliminary clinical efficacy of a vaccine against extraintestinal pathogenic Escherichia coli in women with a history of recurrent urinary tract infection: a randomised, single-blind, placebo-controlled phase 1b trial. Lancet Infect. Dis. 17: 528–537. 28238601 10.1016/S1473-3099(17)30108-1

[59] Levine, M. M., K. L. Kotloff, E. M. Barry, M. F. Pasetti, M. B. Sztein. 2007. Clinical trials of *Shigella* vaccines: two steps forward and one step back on a long, hard road. Nat. Rev. Microbiol. 5: 540–553. 17558427 10.1038/nrmicro1662 PMC3771495

[60] MacLennan, C. A., J. Stanaway, S. Grow, K. Vannice, A. D. Steele. 2023. *Salmonella* combination vaccines: moving beyond typhoid. Open. Forum. Infect. Dis. 10: S58–S66. 37274529 10.1093/ofid/ofad041 PMC10236507

